# Clinical Outcome of Patients with Complete Pathological Response to Neoadjuvant Chemoradiotherapy for Locally Advanced Rectal Cancers: The Indian Scenario

**DOI:** 10.1155/2014/867841

**Published:** 2014-12-28

**Authors:** Snita Sinukumar, Prachi Patil, Reena Engineer, Ashwin Desouza, Avanish Saklani

**Affiliations:** ^1^Department of Surgical Oncology, Tata Memorial Hospital, Dr. Ernest Borges Road, Parel, Mumbai, Maharashtra 400012, India; ^2^Department of Gastroenterology, Tata Memorial Hospital, Dr. Ernest Borges Road, Parel, Mumbai, Maharashtra 400012, India; ^3^Department of Radiation Oncology, Tata Memorial Hospital, Dr. Ernest Borges Road, Parel, Mumbai, Maharashtra 400012, India; ^4^Department of Surgical Oncology, Robotic and Colorectal Surgery, Tata Memorial Hospital, Dr. Ernest Borges Road, Parel, Mumbai, Maharashtra 400012, India

## Abstract

*Introduction*. Neoadjuvant chemoradiotherapy and total mesorectal excision are considered the standard treatment for locally advanced rectal cancer. Various studies have reported pathological downstaging and a complete pathological response rate of 15%–27% following neoadjuvant chemoradiotherapy which has translated into improved survival. We endeavour to determine the clinical outcome of patients attaining a complete pathological tumor response following neoadjuvant chemoradiotherapy in the Indian setting where most of our patient population is younger and presents with aggressive tumor biology. *Materials and Methods*. Clinicopathological and treatment details were recorded for 64 patients achieving pathological complete response from 2010 to 2013. Disease-free survival (DFS), overall survival (OS), and locoregional and systemic recurrence rates were evaluated for these patients. *Results*. After a median follow-up of 30.5 months (range 11–59 months), the 3-year overall survival (OS) was 94.6% and the 3-year disease-free survival (DFS) was 88.5%. The locoregional and systemic recurrence rates were 4.7% and 3.1%, respectively. *Conclusion*. In the Indian subcontinent, despite younger patients with aggressive tumor biology, outcome in complete responders is good.

## 1. Introduction

Total mesorectal excision coupled with neoadjuvant chemoradiotherapy is currently considered the standard treatment for patients with locally advanced rectal cancers (LARC). This multimodality treatment has resulted in improved local control rates, albeit showing no long-term survival benefits [1–5]. Various studies across literature have reported pathological downstaging and a complete pathological response rate (ypCR) of 15%–27% following neoadjuvant chemoradiotherapy (NACTRT) prior to radical surgery [6]. This has translated into not only a superior and improved survival but also decreased locoregional and systemic recurrence.

Through our study we endeavour to determine the clinical outcome of patients attaining a complete pathological tumor response following neoadjuvant chemoradiotherapy and radical rectal surgery in the Indian setting where most of our patient population is younger and presents with aggressive tumor histology.

## 2. Materials and Methods

Four hundred and thirty patients of LARC (cT3, cT4, N+) underwent surgical resection following NACTRT from 2010 to 2013 at the Tata Memorial Hospital. Of the 430 patients, sixty-four patients achieved complete pathological tumor response. Information on demographic data, stage at presentation, and treatment administered were recorded from a prospectively maintained database for these 64 patients. A colonoscopy/sigmoidoscopy was performed on all patients and a tissue diagnosis with biopsy was obtained prior to commencement of treatment. Staging investigations were comprised of a contrast enhanced CT scan of the thorax and a MRI of the pelvis or CT scan of the pelvis prior to receiving neoadjuvant chemoradiotherapy. After completion of NACTRT and prior to surgery all patients were imaged with a pelvic MRI. Patients were staged according to the UICC TNM 2010 classification for rectal cancer [7]. Patients received long course radiation between 40 Gy and 50 Gy in 25#–28# for 5 weeks with twice daily Capacetabine 825 mg/m^2^ concurrently with radiation, as part of NACTRT protocol. All patients were discussed in a multidisciplinary tumor board where treatment decisions were made. Disease-free survival (DFS), overall survival (OS), and locoregional and systemic recurrence rates were evaluated for these 64 patients. DFS was determined from the date of starting treatment, that is, neoadjuvant chemoradiotherapy, to the date of first locoregional or systemic relapse. Overall survival (OS) was calculated from the date of starting treatment, that is, neoadjuvant chemoradiotherapy, to the date of death or last follow-up. Kaplan-Meier curves were calculated to determine survival and log-rank test was used to compare survival outcomes between different subgroups of patients.

## 3. Results

**Figure 1:**
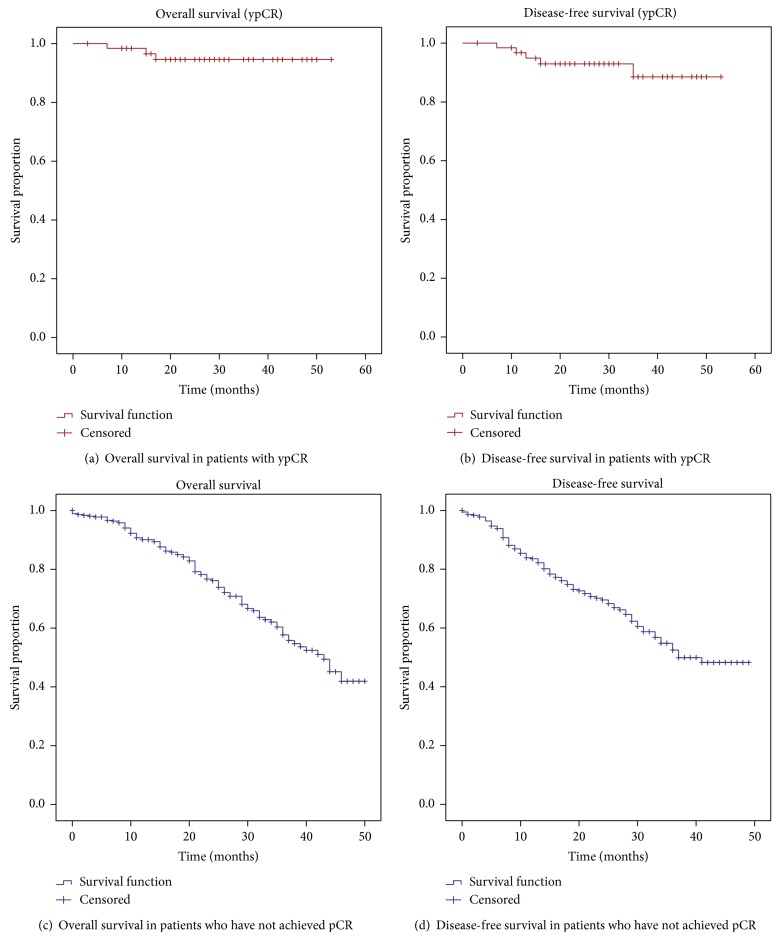
(a) and (b) Survival in patients with a complete pathological response (ypCR). (c) and (d) Survival in patients who have not achieved a complete pathological response.

Clinicopathological characteristics of this cohort are summarized in Table 1. In our study, 65.6% of our patients were male. The median age of presentation was 47 years (range 18–77 years). The median pretreatment carcinoembryonic (CEA) tumor marker level was 3.2 (range 1.1–127 ng/mL). 95.7% received long course radiation as part of NACTRT. The median interval between completion of NACTRT and surgery was 56 days (range 12–206 days). Abdominoperineal resection (APR) was the most common surgical procedure performed. Adjuvant chemotherapy was administered in 60.9% patients. 14.8% patients had a complete pathological tumor response rate (ypCR) with a TRG score of 1/5 (the Mandard scoring system was used) [8]. The commonest histology was classical adenocarcinoma. Signet cell carcinoma and mucinous carcinoma were seen in 14.06% and 7.8% of cases, respectively. The median nodes dissected at surgery were 8 (range 1–21). After a median follow-up of 30.5 months (range 11–59 months), the 3-year overall survival (OS) was 94.6% and the 3-year disease-free survival (DFS) was 88.5% (Figures 1(a) and 1(b)). In patients without complete pathological response, the 3 year OD was 57% and DFS was 52% (Figures 1(c) and 1(d)). The locoregional recurrence rate and systemic recurrence rate were 4.7% and 3.1%, respectively.

## 4. Discussion

The combination of NACTRT and meticulous radical surgery has contributed to increased local control in patients with advanced rectal cancers. Despite this multimodality treatment, the reported 5-year survival in patients with locally advanced rectal cancer is 45%–75% with recurrences occurring in 5%–15% of patients [9]. In India, however, survival remains low, the reported 5-year survival being between 30 and 40% [10, 11]. There is, however, a cohort of patients who achieve complete pathological response following NACTRT and definitive surgery. Several studies across literature have reported a ypCR rate of 15%–27% and a favourable survival in these patients. In a retrospective cohort study of 725 patients, oncologic outcomes after preoperative chemoradiotherapy and radical resection for locally advanced rectal cancer correlated with pathological complete response. This study reported no local recurrences and a distal metastatic rate of 7% in patients achieving a complete pathological response [12].

In our study 14.8% of patients achieved a ypCR (64/430). Our study showed that this cohort of patients with pathological complete response enjoyed a good overall survival as well as disease-free survival. In a recent systematic review and meta-analysis evaluating the clinical outcome of complete pathological response following neoadjuvant chemotherapy in 3363 patients, the reported five-year overall and disease-free survival rates were 90.2% and 87.0%, respectively. A local recurrence rate of 0.7% with a distant failure rate of 8.7% was also reported in this review. This meta-analysis concluded that a complete pathological response following NACTRT was associated with excellent long-term survival, with low rates of local recurrence and distant failure [13]. Capirci et al. in their largest published experience of patients with a pathological complete response following neoadjuvant chemoradiotherapy and radical rectal surgery reported a local failure rate of 0.9% with distant failure occurring in 8.9% [14]. Maas and coworkers in a comparative analysis of pooled individual data of 3105 patients to determine the oncological outcomes for patients with and without a pathological complete response (ypCR) after neoadjuvant chemoradiotherapy showed a 5-year DFS rate of 83.3% in patients with a ypCR as compared with 65.6% for incomplete responders. Five-year local recurrence rates reported were 2.8% versus 9.7% and the rate of distant metastasis was 11.2% versus 25.2% [6]. The findings of our study are in concordance with those published in the literature. In our patients with ypCR the 3-year OS and DFS were 94.6% and 88.5%, respectively. We reported a locoregional and systemic recurrence rate of 4.7% and 3.1%, respectively. This holds true despite a high proportion of our tumors presenting with extracellular mucin (37.5%) and showing aggressive histology (signet cell 14.06%, mucinous 7.8%).

Despite achieving a complete response in the primary tumor, approximately 6.6%–17% of tumor specimens have residual disease in the mesorectal nodes (ypT0N+) [15–17]. Several studies have emphasized the prognostic significance of residual nodal disease [17–19]. Yeo and colleagues in their study of 333 LARC patients concluded that patients achieving a pathological complete response after preoperative NACTRT enjoyed favorable long-term outcomes. They also showed that the most significant independent prognostic factor for DFS and OS, even after total regression of the primary tumor by preoperative NACTRT, was residual nodal disease [15]. Evidence in the literature has shown that ypCR is associated with improved survival. One of the most studied strategies to improve ypCR is the optimum interval between NACTRT and surgery. Several studies have shown that a minimum duration of 6–8 weeks between NACTRT and surgery improves downstaging and increases the chances of a ypCR [20–23]. In our study, the median interval between NACTRT and surgery was 56 days (range 12–127 days).

In our study 60.9% of patients achieving a pathological complete response received postoperative adjuvant chemotherapy. There is however a lack of consensus about the benefit of adjuvant chemotherapy in patients achieving a ypCR. A few studies have shown an improved survival in patients with pathological downstaging and postoperative chemotherapy [15, 24, 25], while others have suggested that adjuvant chemotherapy be administered only in patients with residual nodal disease [26].

In light of the available evidence in the literature, the clinical ramifications of such a pathological complete response could be manifold. There is no doubt that patients achieving a ypCR enjoy improved survival. Hence efforts need to be made to increase the rates of ypCR. This becomes imperative especially in context of the Indian population as majority of our patients are younger and present with an aggressive histology, that is, mucinous and signet cell carcinoma (approx. 7.8% and 14.06%, resp.) [Dr. A Saklani, Tata Memorial Hospital]. Efforts to improve ypCR rates with the addition of oxaliplatin or irinotecan [27, 28] or targeted agents [29, 30] have been disappointing. The integration of translational research and gene expression profiling may assist in identifying factors that may predict tumor response and guide in achieving a higher proportion of ypCR.

## 5. Conclusion

In the Indian scenario, despite younger age and higher proportion of mucinous and signet cell tumors, outcome in complete responders is good and is in concordance with world literature. Efforts need to be made to increase complete response rates in order to avail our patients with maximum benefits in terms of survival and local control.

## Figures and Tables

**Table 1:** Demographic and clinicopathological characteristics.

Characteristics	*n* = 64 (%)
Gender	
Male	42 (65.6%)
Female	22 (34.4%)
Age (yr)	
Range	18–77
Median	47
Stage at presentation^a^	
cT3N0	39 (60.9%)
cT4N0	2 (3.1%)
cT3N+^x^	23 (35.9%)
Distance from anal verge (AV)^b^	
AV-2 cms	25 (39.1%)
2–5 cms	15 (23.4%)
5–10 cms	20 (31.3%)
>10 cms	04 (6.3%)
Pretreatment CEA (ng/mL)^c^	
Range	1.1–127
Median	3.2
Surgery	
Abdominoperineal resection (APR)^d^	29 (45.3%)
Low anterior resection	10 (15.6%)
Anterior resection	22 (34.3%)
Intersphincteric resection	03 (4.6%)
Histology	
Classical adenocarcinoma	50 (78.1%)
Mucinous adenocarcinoma	5 (7.8%)
Signet cell adenocarcinoma	9 (14.06%)
Extracellular mucin pool	
Yes	24 (37.5%)
No	40 (62.5%)
Total nodes dissected	
Range	1–21
Median	08
Radiotherapy type	
Long course	67 (95.7%)
Short course	03 (4.3%)
Radiotherapy dose	
50 GY	48 (75%)
40–45 GY	14 (22%)
25 GY	02 (3.1%)
Laparoscopy	
Yes	11 (17.2%)
No	53 (82.8%)
Interval of NACTRT to surgery (days)	
Range	12–206
Median	56.5
Postoperative chemotherapy	
Yes	39 (60.9%)
No	25 (39.1%)

^a^Clinicoradiological staging.

^b^AV: anal verge.

^c^CEA: carcinoembryonic antigen.

^d^APR indicates abdominoperineal resection.

^x^N+: positive nodal status.
